# Comparative analysis of inflammatory bowel disease (IBD) patient- and service-reported quality of care using 2019 and 2023 UK benchmarking data from more than 26 000 adult patient respondents and 154 IBD services

**DOI:** 10.1093/ecco-jcc/jjag005

**Published:** 2026-01-23

**Authors:** A Barney Hawthorne, Paul Christiansen, Ian Arnott, J R Fraser Cummings, Liz Dobson, Alexandra Kent, Jimmy K Limdi, Robert J Mulligan, Gareth C Parkes, Fiona Rees, Christian P Selinger, Jessica Turner, Nathaniel Woo, Lisa Younge, IBD Registry, Contributors to the IBD UK Task & Finish group,, Katie Adams, Rachel Ainley, Ian Arnott, Graham Bell, Kathleen Bone, Seema Buckley, Angela Chana, J R Fraser Cummings, Shahida Din, Liz Dobson, Fiona Eldridge, Jonathan Evans, Melissa Ganendran, Wendi Harrison, A Barney Hawthorne, Katie Keetarut, Alexandra Kent, Jon Kwok, Christopher A Lamb, Jimmy K Limdi, Charles Maxwell-Armstrong Gayle Martin, Gareth C Parkes, Emma Pryde, Nabil Quraishi, Marianne Radcliffe, Fiona Rees, Jenna Robinson, Ruth Rudling, Christian P Selinger, Esha Sharma, Sarah Sleet, Ben Skipp, Anwen Thomas, Jess Turner, Ruth Wakeman, Catherine Winsor, Sarah York, Lisa Younge, IBD Service Survey Collaborators, Mostafa Afifi, Lucy Aitchison, Gill Anderson, Gillian Bain, Zahra Bayaty, Pauline Bell, James Berrill, Sophia Bishop, Paul Blaker, Keith Bodger, Dulini Broomhall, Victoria Burn, Jeffrey R Butterworth, Vida Cairnes, Rachel Campbell, Julie Carris-Wright, Rowena Castillo, Louise Caulfield, Alex Cheshire, Daljit Chohan, Marion Clark, Katie Clark, Carol Cobb, Josephine Coe, Rachel Cooney, Lourdes Cumlat, Patricia Daly, Anups De Silva, Anjan Dhar, Benjamin Disney, Fiona Donovan, Helen Empson, Bridgette Fraser, Aileen Fraser, Becky George, Nivedita Ghosh, Lynn Gray, James Gulliver, Anton Gunasekera, Markus Gwiggner, Sarah Harrison, Wendi Harrison, Virginia Hay, Caroline Hayhurst, Patricia Hooper, Louise Horne, Hasnain Jafferbhoy, Kerrie Johns, Emma Johnston, Karen Kemp, Dawn Kilpatrick, Matthew Kirkbride, Beverley Kirkham, Andrew Kneebone, Ioannis Koumoutsos, Christopher A Lamb, Lianne Lewis, James O Lindsay, Amy Lloyd, Caroline Lock, Helen Ludlow, Clare Macpherson, Anne Macrae, Philip Mairs, Gayle Martin, Joy Mason, Kirsten Mccaul, Gordon Mcfarlane, Ross McGettigan, Vidya Morgan, Deborah Morris, Graham Morrison, Themba Mudege, Ann Muir, Jasbir Nahal, Mark Narain, Ebenezer Nellikunnil, Elizabeth Nelson, Emma Nowell, Arabis Oglesby, Joanne Owens, Hari Padmanabhan, Lauren Laydon Parkin, Jacqueline Paterson, Polychronis Pavlidis, Simon Peake, Mohammad Farhad Peerally, Kathryn Phillis, Tim Raine, Maxine Rawle, Shuvra Ray, Ian Reilly, Kerry Robinson, Jenna Robinson, Matthew D Rutter, Linda Samuel Tuck, Glyn Scott, Shaji Sebastian, Christian P Selinger, Caroline Sharratt, Ian Shaw, Jayne Slater, Melissa A Smith, Samuel Smith, Katie Smith, Amudha Somasekar, Sunil Sonwalkar, Seth Squires, Alan Steel, Samuel Sunil, Diarmid Sutherland, Nora Thoua, Diane Upton, Jennifer Veryan, Aldea Waters, Susie Wen, Lydia White, Simon R Whiteoak, Sarra Wilcox, Lisa Younge, Pam Younge, Christopher A. Lamb

**Affiliations:** Department of Gastroenterology, Cardiff & Vale University Health Board, Heath Park, Cardiff, United Kingdom; Department of Psychology, University of Liverpool, Liverpool, United Kingdom; Edinburgh IBD Unit, Western General Hospital, Edinburgh, United Kingdom; Department of Gastroenterology, University Hospital Southampton NHS Foundation Trust, Southampton, United Kingdom; Clinical and Experimental Sciences, Faculty of Medicine, University of Southampton, Southampton, United Kingdom; IBD Registry, London, United Kingdom; Department of Gastroenterology, King’s College Hospital NHS Trust, London, United Kingdom; Division of Gastroenterology-Section of IBD, Northern Care Alliance NHS Foundation Trust, Manchester, United Kingdom; Manchester Academic Health Sciences, University of Manchester, Manchester, United Kingdom; Translational & Clinical Research Institute, Newcastle University, Newcastle upon Tyne, United Kingdom; Department of Gastroenterology, Newcastle upon Tyne Hospitals NHS Foundation Trust, Newcastle upon Tyne, United Kingdom; Department of Gastroenterology, Royal London Hospital, Barts Health NHS Trust, London, United Kingdom; Department of Gastroenterology, University Hospitals of Sussex NHS Trust, Brighton, United Kingdom; Department of Gastroenterology, Leeds Teaching Hospitals NHS Trust, Leeds, United Kingdom; Leeds Institute of Medical Research, University of Leeds, Leeds, United Kingdom; Crohn’s & Colitis UK, Hatfield, United Kingdom; Crohn’s & Colitis UK, Hatfield, United Kingdom; Department of Gastroenterology, St Mark’s Hospital, Harrow, United Kingdom; Department of Gastroenterology, King’s College Hospital NHS Trust, London, United Kingdom; Translational & Clinical Research Institute, Newcastle University, Newcastle upon Tyne, United Kingdom; Department of Gastroenterology, Newcastle upon Tyne Hospitals NHS Foundation Trust, Newcastle upon Tyne, United Kingdom

**Keywords:** Inflammatory bowel disease, Ulcerative colitis, Crohn's disease, Quality improvement, Benchmarking, Multidisciplinary team, Cost-effectiveness, Service development, Self-management

## Abstract

**Introduction:**

The IBD UK Benchmarking surveys, conducted in 2019 and 2023, collected repeated data regarding the quality of inflammatory bowel disease (IBD) care across the UK using both service self-assessments and patient-reported experience measures (PREMs). We aimed to assess variation between patient and provider perspectives.

**Methods:**

All UK hospitals offering specialist IBD services were invited to complete online surveys. Patients were invited through social media, charities, and clinical services. This study compared changes over the 4 years and examined alignment between healthcare-reported and patient-reported assessments.

**Results:**

From 26 760 patient responses and 154 service assessments, patient-perceived care quality (PPCQ) declined between 2019 and 2023 (*P* < .001). Male sex and older age were associated with higher PPCQ. Greater disease severity was associated with lower PPCQ (*P* < .001). More patients reported IBD symptoms to impact activities of daily living in 2023 (*P* < .001). Factors associated with higher PPCQ included rapid diagnosis, being supported by an IBD team, and having knowledgeable IBD nurses. Access, information, communication, and empowerment were identified by patients as needing improvement (*P* < .001). Services with lowest quartile quality scores in 2019 demonstrated significant improvement over time, whilst those with highest 2019 scores demonstrated significant deterioration in PPCQ (*P* < .001). Services reported better performance than patients (*P* < .001).

**Conclusions:**

These data underscore the importance of assessing lived experience and the care quality perception gap between patients and service providers. Regular benchmarking including PREMs should be used to drive and assess service-level, national and international quality improvement initiatives.

## 1. Introduction

The first iteration of the UK inflammatory bowel disease (IBD) Standards was launched in 2009.^1^ Formed in 2017, IBD UK is a coalition of 16 professional bodies, Royal Colleges, and patient organizations working together to advocate for the delivery of consistent, safe, high-quality personalized care for all affected by IBD, irrespective of demographics or where they live in the UK.^2^ The latest IBD UK Standards, updated in 2019, define best practice in service design and delivery of IBD care across all aspects of the patient experience.^3^ The Standards provide a framework and metrics by which the quality of IBD services can be measured across multiple domains with the goal of improving the standard of IBD care delivery nationally within the National Health Service (NHS) and providing an exemplar model for international quality improvement.

In 2019, IBD UK delivered the first IBD benchmarking survey,^4^ closely aligned with all clinical domains of the IBD UK Standards. This was a parallel exercise simultaneously asking IBD hospital services across all four nations of the UK to self-assess their performance whilst also asking patients to rate the quality of care they experience. Analysis of 2019 data demonstrated the importance of aspects of care to patients that are often under-recognized by healthcare professionals: communication, shared decision-making, information provision, and the role of IBD nurse specialists.^4^ This survey was unique in its size and scope and was designed to drive UK national and local quality improvement prior to planned re-assessment at regular intervals.

The importance of the patient voice, whether by regular collection of patient-reported outcome or experience measures (PROMs or PREMs) or by other surveys for patients with chronic medical conditions,^5^ has been emphasized in the NHS 10-year plan—particularly systematic and comparable collection of data.^6^ This facilitates more truly patient-centered care, drives quality improvement and standardized care, and informs policy-making and research.^7^^,^^8^

The COVID-19 pandemic in 2020 had a profound impact on the NHS,^9^ and on IBD patients and many aspects of their care.^10^ IBD UK repeated the national benchmarking process in 2023, providing an opportunity to determine changes in care provision over this turbulent time and the subsequent “new normal.” A report summarizing the main 2023 findings, “The State of IBD care in the UK,”^11^ was launched in a UK Parliamentary Reception in December 2024.

This is a critical time for the UK NHS and global healthcare systems. The NHS 10-year plan envisages increased digital connectivity, a shift from hospital to community, and a shift from sickness to prevention.^6^ Each of these “pillars” has great potential for managing chronic health conditions, but they require active patient input to ensure that service efficiency does not come at the expense of quality of care for patients. The 2019 patient survey included questions regarding access to information (which should improve with greater digital resources); patient experience of follow-up and whether they were seen as often as they needed; whether access from community follow-up to hospitals when flaring was rapid enough; and whether screening by colonoscopy for colorectal cancer occurred if required. The NHS 10-year plan also emphasizes the strong role for the public in shaping and measuring care quality.^6^

This analysis aims to provide further evidence of the validity of patient-reported service quality, by comparison of adult IBD patient- and service-reported metrics, and determining the degree of agreement between what service providers believe they deliver and the experience of those receiving that care. We also assess factors associated with the changes in quality of care reported between 2019 and 2023.

## 2. Methods

The 2023 benchmarking Patient and Service Surveys were based on the 2019 surveys where detailed methodology has been previously published.^4^ Both were updated for clarity in the light of results from the 2019 surveys, but keeping questions the same wherever possible, to facilitate comparison between 2023 and 2019 results. Survey development for 2023 was undertaken by IBD UK Board Members plus appointed Task and Finish groups. These groups were multidisciplinary including patients, physicians, surgeons, IBD nurse specialists, pharmacists, radiologists, pathologists, and members of staff at Crohn’s & Colitis UK. The Patient Survey development included focus groups of people living with IBD. Previously, questions for adults and children were all within the same survey, but for 2023 benchmarking they were separated into adult and pediatric surveys for patients and for IBD services, then analyzed separately. Objective questions required yes/no responses or selection from a range of options (eg, waiting time periods). For subjective questions a statement was presented with a five-point Likert scale response (Strongly disagree/­Disagree/Neither agree nor disagree/Agree/Strongly agree).

### 2.1. Patient survey

The 2023 surveys were designed for online completion (although patients had an option to request a paper copy or translation if required). As in 2019, patient questions focused on lived experience over the past year, and questions regarding diagnostic pathways, admission to hospital, and surgery were offered if these had been encountered within the past 2 years. There were 86 questions in total across all clinical domains (Table 1): pre-diagnosis, newly diagnosed, flare management, surgery, hospital in-patient care, ongoing care, and monitoring (see IBD UK website: https://ibduk.org/reports/ibd-uk-2023-benchmarking-survey-results). Some questions used branching logic (eg, questions related to surgery were not presented to patients unless they had undergone an operation within the past 2 years). In addition, new questions were asked for 2023: patients were asked to assess the relative importance of their experience of care across six aspects of care (Table 1: access to treatment, information provision, communication, patient empowerment, well-being support, and research), and to rate how much these domains of care needed improvement in their local service. As in 2019, patients were asked to rate the overall quality of their care in the preceding 12 months as “poor,” “fair,”, “good,” “very good,” or “excellent.” For statistical analysis this (and other Likert scales) were converted to a 1–5 scale (1 = poor through to 5 = excellent). The Patient Survey was conducted from April to June 2023 and was promoted by printed flyers and digital methods, particularly social media, emails, and the IBD UK website. In light of the 2019 survey having lower participation from minority groups, a specialist research consulting group visited several services to investigate issues, conduct epidemiological analysis, and develop engagement plans for each minority group. To preserve anonymity, no email or identifiable data were recorded, and there were no free text questions, which might contain identifiable factors. There was an anonymous/personal “return-to-complete-later” ability—this was both to encourage completion and to reduce the risk of people starting a second survey inadvertently. Whilst possible that patients may have submitted the survey twice, this was felt to be very unlikely.

**Table 1.** IBD UK Standards clinical domains and aspects of care assessed by benchmarking service performance through patient feedback.

IBD UK standards clinical domains	Aspects of care assessed through patient feedback
Pre-diagnosis	Access to treatment
Diagnosis	Information provision
Flare	Communication
Surgery	Patient empowerment
Inpatient care	Well-being support
Ongoing care and monitoring	Research

### 2.2. Service survey

Many of the 2019 questions were in tiers of three questions. For 2023, all questions were separated to simplify statistical analysis. There were 121 questions in total (see IBD UK ­website: https://ibduk.org/reports/ibd-u k-2023-benchmarking-survey-results). Comparison of responses between the 2019 and 2023 surveys, and between Patient and Service Surveys, was only made if the questions were the same (other than minor wording alterations) and response options were the same. The Service Survey was conducted in June–August 2023, promoted through IBD UK member organizations, and a database of IBD services. Reminders were sent to those not responding, and services were encouraged to complete the survey as a team of healthcare professionals including physicians, surgeons, nurses, and other multidisciplinary team members as needed. Services each had a unique identifier to ensure that only one survey was analyzed per service. To make completion less onerous, survey responses did not require data collection, but were the perception of the team about each aspect of their survey. They were encouraged to use audit data where already available. For objective questions (eg, waiting times), they were offered a choice of several ranges of time, which were the same as those in the patient survey.

### 2.3. Statistics

Data were analyzed using R (v.4.4.1), IBM SPSS Statistics v.29.0.1.1, and GraphPad Prism (v.10.3.0). The lme4 and lmertest R packages were used to run multilevel models and the clubSandwich package in R was used to calculate cluster-robust variance estimators. The sandwich package was used to calculate these in the non-multilevel linear models. When exploring the impact of participant- and service-level variables on participant care satisfaction, multilevel models were fitted, as participants were nested within services necessitating a random intercept for service. Patient-level variables included age, sex, type of IBD, whether diagnosed within the past 2 years, and condition severity. Service-level variables identified key features (size, staffing levels, organizational factors such as whether IBD service had a leadership team, and were led by a named gastroenterologist, and waiting times for key events). Due to the large number of patient questions, those analyzed were selected to represent different aspects of care (access to treatment, information provision, communication, empowerment, well-being support and research). Many question responses fell into clusters and, for these groups, the question with the strongest correlation was used. Questions that were unchanged between the 2019 and 2023 surveys were used for comparative analysis, and likewise those where question and response options were the same were used for comparison between patient and survey data. To ensure the utility of the random intercept, a null linear model with no random intercept was compared to a null linear model with a random intercept, and differences in the log-likelihood of the two models were assessed with a χ^2^ test and by differences in the Akaike information criterion (AIC) values (AIC differences >10 were deemed to be a substantial difference). Intraclass correlation (ICC) was used to ascertain the amount of variance in care perception attributable to the service level. Patient- and service-level predictors were added to the models. The amount of variance these variables predicted at both the patient and service level was calculated. These analyses required response to all variables, and excluded patients not linked to a service. The large survey size meant that adequate numbers would remain for analysis.

### 2.4. Data handling

Study data were collected and managed using REDCap electronic data capture tools^12^ hosted at the IBD Registry. The IBD Registry worked on the design and delivery of the surveys, collection of data as well as acting as data controller. The data were shared with Crohn’s & Colitis UK for analysis under a data sharing agreement. All patient data were collected and analyzed in anonymous form and only collated data are presented. Service identifiers were removed before analysis and presentation.

## 3. Results

**Figure 1.**
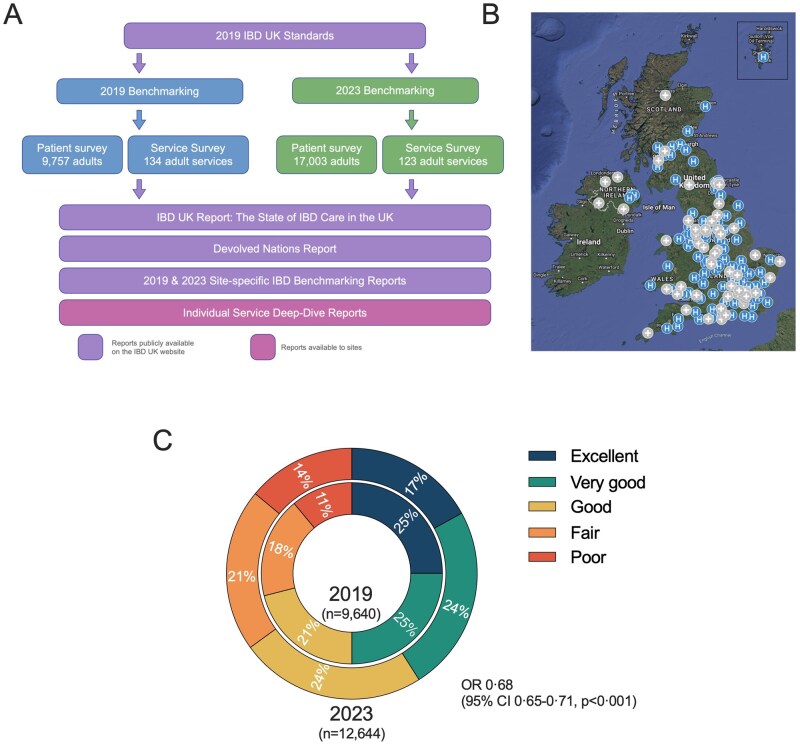
(A) Overview of the 2019 and 2023 IBD UK benchmarking processes including number of Patient Surveys and Service Surveys submitted in each. State of IBD Care in the UK, Devolved Nations, and Site-Specific Reports are available publicly at https://ibduk.org/reports. Individual Service Deep-Dive Reports have been supplied to each participating service to support quality improvement (QI) initiatives. (B) UK map with 2023 responding (blue H) and non-responding (grey +) adult IBD services. (C) Adult patient-perceived care quality (PPCQ) reported in the 2019 (*n* = 9640 respondents) and 2023 (*n* = 12 644 respondents) benchmarking surveys. Patient respondents were requested to rate their care over the previous year as excellent, very good, good, fair, or poor. Map data ©2026 GeoBasis-DE/BKG (©2009), Google Imagery ©2026 NASA.

The 2019 benchmarking data included 9757 adult Patient Survey responses and 134 Service Survey responses. The 2023 Patient Survey included 17 003 adult responses, and Service Survey responses included 123 adult services (Figure 1) across the whole of the UK, with responses from England, Scotland, Wales, and Northern Ireland (Table 2). Of the 177 adult IBD services in the UK, 123/177 (69%) completed the 2023 survey. Patients were asked to state the hospital or service where they were being treated, with a median of 53 (interquartile range [IQR] 23-85) respondents per service. In total, 10 063 patients attended a service that submitted a 2023 Service Survey, 1634 recorded a service that had not submitted a survey, and for 5307 no hospital was recorded by the patient. In aggregate, 154 unique services responded in one (33.1%) or both (66.9%) of the 2019 and 2023 surveys.

**Table 2.** Demographics of the 2019 and 2023 IBD adult Patient Survey respondents.

	Category	2019 (*n* = 9757)	2023 (*n* = 17 003)	Change from 2019 to 2023: χ^2^, (degrees of freedom), *P*-value
**Diagnosis**	Crohn’s disease	5013/9756 (51%)	8192/17 003 (48%)	79.3, (3), *P* < .001
	Ulcerative colitis	4358/9756 (45%)	7740/17 003 (46%)	
	IBD unclassified	283/9756 (2.9%)	725/17 003 (4.3%)	
	Other	102/9756 (1%)	342/17 003 (3.8%)	
**Age (years)**	<18	100/9745 (1%)	33/16 988 (0.2%)	523, (7), *P* < .001
	18-24	830/9745 (8.5%)	883/16 988 (5.2%)	
	25-34	1983/9745 (20%)	2734/16 988 (16%)	
	35-44	1897/9745 (20%)	2893/16 988 (17%)	
	45-54	2072/9745 (21%)	2971/16 988 (18%)	
	55-64	1561/9745 (16%)	2968/16 988 (18%)	
	65-74	1039/9745 (11%)	1941/16 988 (11%)	
	≥75	263/9745 (2.7%)	676/16 988 (4.0%)	
**Self-reported gender**	Female	6458/9705 (67%)	10 864/15 117 (72%)	79.4, (1), *P* < .001
	Male	3247/9705 (34%)	4253/15 117 (28%)	
**Ethnic group**	White	9019/9623 (94%)	14 366/14 991 (96%)	15.7, (3), *P* < 0.001
	Asian	186/9623 (1.9%)	297/14 991 (2.0%)	
	Black	23/9623 (0.023%)	61/14 991 (0.41%)	
	Other^a^	395/9623 (4.1%)	267/14 991 (1.8%)	
**Date of diagnosis**	>2 years ago	7832/9713 (81%)	13 073/16 304 (80%)	0.38, (1), *P* = .38
	≤2 years	1881/9713 (19.4%)	3231/16 304 (20%)	
**Overall patient-perceived care quality**	Excellent	2390/9183 (26%)	2201/12 644 (17%)	259, (4), *P* < .001
	Very good	2259/9183 (25%)	2990/12 644 (24%)	
	Good	1896/9183 (21%)	2989 (24%)	
	Fair	1618/9183 (18%)	2650 (21%)	
	Poor	1020/9183 (11%)	1814/12 644 (14%)	
**How would you generally describe the severity of your IBD over the last 3 months?**	No symptoms, not affected	1422/9726 (15%)	1583/15 053 (11%)	527, (4), *P* < .001
	Symptoms did not get in the way of activities	2902/9726 (30%)	3808/15 053 (25%)	
	Symptoms got in the way of some activities	2552/9726 (26%)	5996/15 053 (40%)	
	Symptoms significantly got in the way of everyday activities	2020/9726 (20%)	2809/15 053 (19%)	
	Unable to do everyday activities	830/9726 (8.5%)	857/15 053 (5.7%)	
**UK nation**	England	8080/9753 (83%)	14 193/16 998 (84%)	52, (3), *P* < .001
	Scotland	768/9753 (7.9%)	1500/16 998 (8.8%)	
	Wales	521/9753 (5.3%)	851/16 998 (5.0%)	
	Northern Ireland	384/9753 (3.9%)	454/16 998 (2.7%)	

Total numbers of surveys are shown at head of columns, with denominators for each question reduced to account for non-responders.

a Ethnic group category “Other” includes: mixed (White and Asian), mixed (White and Black Caribbean), mixed (White and Black African), mixed (any other mixed background), other ethnic groups (Chinese), and any other ethnic group.

### 3.1. Adult patient-perceived care quality in 2019 and 2023

Patient-perceived care quality (PPCQ) in 2019 and 2023 is shown in Figure 1 and Table 2. As shown in the National Report,^11^ PPCQ fell between 2019 and 2023 with the percentage rating their care as either excellent, very good, or good falling from 71% to 65% (*P* < .001). Of 11 697 patient responders with complete data, 86% (10 059) had an IBD service that completed a survey, with a mean PPCQ (on a scale from 1 [poor] to 5 [excellent]) of 3.32. The PPCQ was lower for the 14% (1638) of patients whose centers did not respond to the IBD Service Survey: 3.08 (*P* = .007). There were similar findings in 2019.

### 3.2. Association of patient factors with PPCQ

Patient characteristics (Table 2) changed significantly between the two Patient Surveys. Crohn’s disease patients accounted for a smaller percentage: (48% [8192/17 003]) of the overall cohort in 2023, and there were more patients aged 55 years or more (37.1% [5585/16 988]). The 2019 results showed more women responded than men, but this had become even more marked in 2023, with 72% (10 864/15 117) female respondents. Compared to 2019, the underrepresentation of ethnic minority patients was similar in 2023, comprising only 4.2% (625/14 991) of respondents. Twenty per cent (3231/16 304) of respondents were diagnosed in the 2 years prior to the 2023 Patient Survey, very similar to 2019.

In a regression model of factors affecting PPCQ (Table 3), older participants and males had more positive PPCQ. Those with greater disease severity (measured by impact on daily activities as shown in Table 2) had lower PPCQ. Patient characteristics accounted for approximately 12% of the participant-level variance in PPCQ, which was 93%. Services accounted for approximately 7% of the variance in PPCQ (ICC = 0.067, *P* < .001), with 19% of this variance (ie, 1.3% overall) related to patient characteristics.

**Table 3.** Predictors of 2023 patient-perceived care quality (PPCQ).

	B	SE	*P*-value	95% CI
Primary diagnosis (CD)^a^	0.039	0.064	.110	−0.086 to 0.009
Age category	0.065	0.009	<.001	0.047 to 0.0816
Sex (male)^b^	0.176	0.026	<.001	0.124 to 0.228
Diagnosed in the last 2 years (Yes)^c^	0.049	0.034	.154	−0.019 to 0.117
Disease severity	−0.418	0.012	<.001	−0.442 to −0.395

*n* = 11 958 patients.

Abbreviations: SE, standard error; CI, confidence interval; B, beta coefficient (the estimated change in the dependent variable [PPCQ] in response to a 1-unit change in predictor variable while all other predictors are constant); CD, Crohn’s disease.

a Results show the effect of CD in comparison to UC.

b Male compared to female.

c Yes compared to no.

### 3.3. Association of patient and service responses to PPCQ

To explore the relationship of both patient response and service response predictors to PPCQ, a multilevel model was run with service as a random intercept (Table 4). Patient factors were included in the model. The model with predictors was significantly better than the random intercept-only model (*P* < .001) and accounted for approximately 89% of the service-level variance and 49% of the participant-level variance. Significant independent predictors of PPCQ from the Patient Survey were time from referral to diagnosis from their first hospital appointment, having knowledgeable IBD nurses, and being supported by an IBD team.

**Table 4.** Multilevel, multivariable regression model of Patient factors, and Patient and Service Survey responses associated with patient-perceived care quality (PPCQ).

	Result^a^	B	SE	*P*-value	95% CI		
**Patient factors**							
Primary diagnosis (CD)^b^	See Table 2	−0.14	0.06	.048	−0.28	to	−0.001
Age category		0.085	0.02	<.001	0.05	to	0.12
Sex (Male)^c^		0.10	0.07	.15	−0.04	to	0.25
Worsening disease severity		−0.22	0.04	<.001	−0.29	to	−0.15
							
**Patient responses**							
Time from referral to first hospital appointment	1-3 months	−0.07	0.03	.03	−0.13	to	−0.01
Time from diagnosis to start of treatment	2-7 days	−0.04	0.02	.08	−0.09	to	0.01
Named gastroenterologist responsible for care (Yes)^d^	86.9%	−0.14	0.06	.03	−0.25	to	−0.02
IBD nurses are knowledgeable	83.8%	0.11	0.05	.04	0.00	to	0.21
Offered research opportunity in past year (Yes)^d^	4.7%	−0.15	0.09	.13	−0.34	to	0.05
Supported by IBD team to manage my condition	Agree	0.64	0.04	<.001	0.56	to	0.71
							
**Service responses**							
Leadership team of senior clinician, nurse, and manager (Yes)^d^	81.7%	0.09	0.09	.35	−0.10	to	0.28
Service led by named gastroenterologist (Yes)^d^	91.6%	−0.21	0.14	.16	−0.54	to	0.11
Tertiary service (Yes)^d^	27.9%	0.11	0.08	.17	−0.05	to	0.26
No. of consultant gastroenterologists with IBD interest (whole time equivalent)	3.25	0.01	0.02	.38	−0.02	to	0.05
No. of IBD specialist nurse IBD sessions (whole time equivalent)	3.0	−0.01	0.03	.84	−0.06	to	0.05
Frequency of multidisciplinary team meetings	Weekly	0.02	0.05	.66	−0.08	to	0.13
Wait time from referral to hospital appointment	1-3 months	0.00	0.03	1.00	−0.06	to	0.06
Wait time for treatment after diagnosis	2-7 days	−0.03	0.05	.56	−0.13	to	0.07
Wait time for elective surgery	4-8 weeks	−0.04	0.02	.13	−0.08	to	0.01
Patients are recruited to research studies	Agree	0.05	0.03	.13	−0.02	to	0.12
Patients involved in service development	Neither agree nor disagree	−0.04	0.03	.12	−0.10	to	0.01

*n* = 870 patients with complete data, *n* = 126 IBD services.

Abbreviations: CD, Crohn’s disease; SE, standard error; CI, confidence interval; B, beta coefficient (estimated change in dependent variable [PPCQ] in response to a 1-unit change in predictor variable while all other predictors are constant).

a Where not shown as a percentage, results are median values for numbers, Likert scale, or range of waiting time.

b Results show effect of Crohn’s disease response compared to ulcerative colitis.

c Effect of male compared to female.

d Effect of yes compared to no.

Three nurse-related responses had been explored in this model by adding each separately. Having knowledgeable IBD nurses and being seen by an IBD nurse on admission for surgery were independent predictors of PPCQ, but a general question regarding having access to an IBD nurse did not achieve significance. IBD specialist nurses have a key role in providing access for patients, as shown by the route patients report using when having a flare. Of 8538 respondents, 41.4% reported contacting the IBD nurse initially, with the next most common route being a gastroenterologist (4.7%). Self-management was reported by 32.8%. None of the Service Survey response predictors were significant.

#### 3.3.1. Patient assessment of value of different aspects of care delivery in 2023

**Figure 2.**
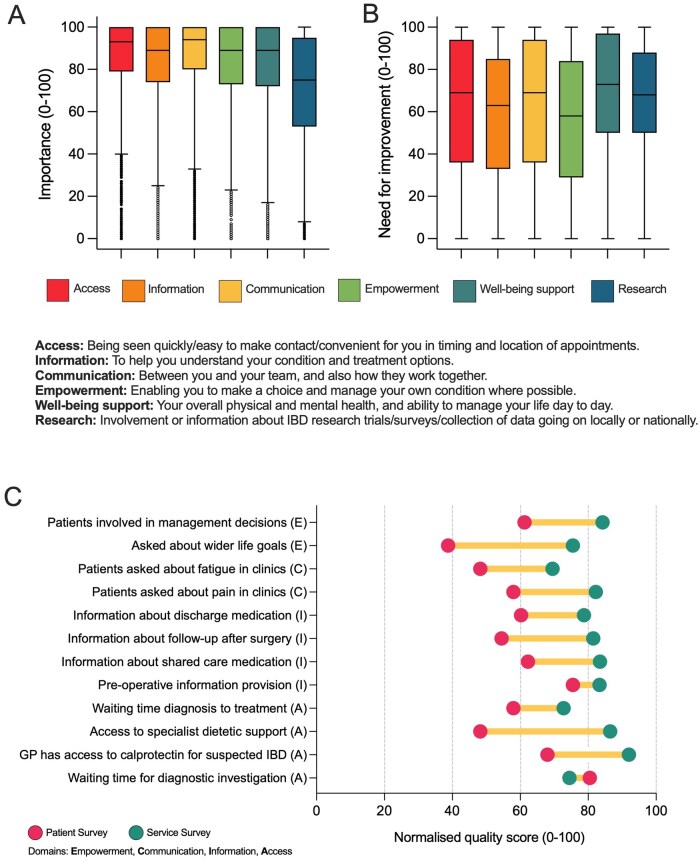
Adult perception of (A) importance and (B) perceived need for improvement across six aspects of the care they experience. Respondents rated aspects on a scale of 0-100. Boxes depict median and interquartile range, whiskers the 1-99 centile, and circles outliers. Response number was median (range) 13 562 (12 760-14 595). (C) Comparative patient- and service-reported quality of care across aspects of care. E = empowerment; C = communication; I = information; A = access; number of Patient Survey respondents for each question was median (range) 8561 (732-15 645); Service Survey response number was median (range) 123 (117–126). All differences were significant (*P* < .001).

In 2023 patients were asked to rate the importance to them of the experience of six aspects of care (Figure 2A). Median importance values were high across all aspects, with patients ranking communication (94/100) and access to care (93/100) highest, followed by information provision, empowerment, and well-being support (each 89/100), and research (75/100). They were also asked to rate how much improvement was needed for these six aspects of care in their own service (Figure 2B). The highest median need values were for well-being support (73/100) followed by communication and access to care (each 69/100), then research (68/100), information provision (63/100), and empowerment (58/100).

### 3.4. Variation between patient- and service-reported data in the 2023 survey

Where questions and response options between the Patient Survey and Service Survey were comparable, IBD service responses were compared with patient responses within services in order to assess for alignment or disparity between service-reported metrics, and those receiving care. As shown in Figure 2C, all but one service responses were more positive than the mean patient responses, and this was the same across different aspects of care (empowerment, communication, information, and access). Similarly a “yes/no” question about provision of a personalized care plan showed the same direction of difference, reporting “yes” by 6.7% of 14 671 patients vs 26% of 123 clinical services. The only exception was the waiting time for diagnostic investigations with patients reporting slightly shorter waiting times than IBD services. The largest discrepancy was on access to specialist dietetic support with services having a normalized score of 86, compared to a patient score of 48.

### 3.5. Change in PPCQ between 2019 and 2023

**Figure 3.**
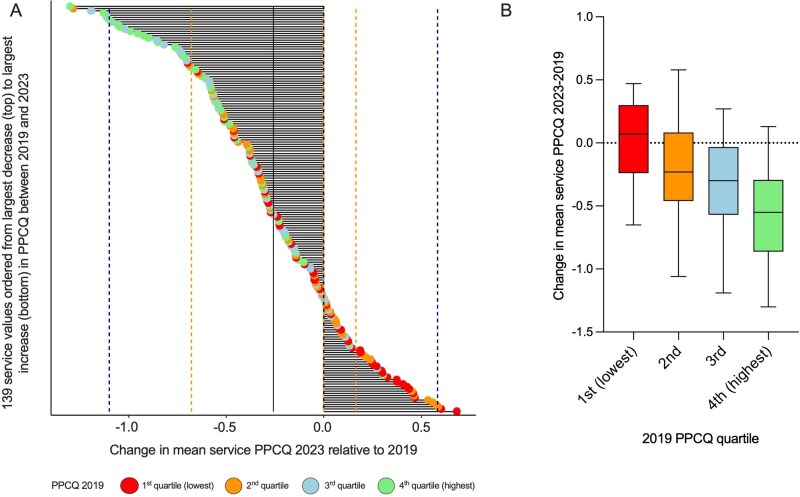
Change in patient-perceived care quality (PPCQ) between 2019 and 2023. (A) For 139 services, horizontal bars show the change in mean PPCQ (2023-2019) ordered by size of change. Solid and dotted vertical lines represent mean change and one and two standard deviations from the mean. Colored circles represent which quartile the IBD service ranked in 2019 for PPCQ. Quality is reported on a five-point scare (poor = 1, through adequate, good, very good, to excellent = 5). (B) Change in mean service PPCQ (2023-2019) for each quartile of 2019 PPCQ values.

For each service with both 2019 and 2023 data, the changes in mean PPCQ values between the two surveys are shown in Figure 3A. The median number of patient responders per site was 60 (IQR 37–88). Patient data were included where their IBD service had not completed a Service Survey, explaining the larger number of centers (*n* = 139) included. There was no significant association between respondent numbers and change in score between 2019 and 2023. The overall mean difference was a fall of 0.264 (95% confidence interval 0.195-0.334, *P* < .001), falling from 3.29 (between good and very good) in 2019 to 3.02 in 2023. Those centers whose mean PPCQ in 2019 was higher were significantly more likely to have a deterioration over the next 4 years, whilst the opposite applied for those with worse values in 2019, who were more likely to have improved PPCQ (*P* < .001) (Figure 3B).

### 3.6. Impact of service factors on PPCQ change from 2019 to 2023

A linear regression model was used to understand whether service factors were predictive of change in mean service PPCQ scores from 2019 to 2023. Mean service PPCQ in 2019 was added as a predictor in the model (to account for regression to the mean and avoid spurious associations). Service factors shown in Table 5 were added as predictors. The overall model accounted for approximately 39% of variance in change scores (*P* < .001). The only service factor that was associated with a change in PPCQ was whether the service was led by a named gastroenterologist. It is of note that the type of service (secondary or tertiary), the level of multidisciplinary staffing, and the number of IBD patients cared for by a service did not contribute to the change in PPCQ in this model.

**Table 5.** Multivariable regression model of predictors of changes in service patient-perceived care quality (PPCQ).

	Result^a^	B	SE	*P*-value	95% CI
**Care perception 2019**	Good^b^	−0.62	0.074	<.001	−0.77 to −0.4875
**IBD Service completed survey (Yes)** ^c^	85.4%	−0.05	0.069	.45	−0.19 to 0.084
**Led by a named gastroenterologist (Yes)** ^c^	91.6%	0.25	0.12	.034	0.020 to 0.49
**Tertiary service (Yes)** ^c^	27.9%	−0.091	0.071	.21	−0.23 to 0.051
**Number of IBD patients (1000s)**	2000	<0.001	<0.001	.37	−0.001 to 0.001
**Leadership team of physician, specialist nurse, and manager (Yes)** ^c^	81.7%	0.076	0.089	.40	−0.10 to 0.25
**Consultant IBD gastroenterologists WTE**	2.8	0.012	0.009	.18	−0.006 to 0.029

*n* = 139 services.

Abbreviations: WTE, whole time equivalent; SE, standard error; CI, confidence interval; B, beta coefficient.

a Results shown as percentage yes or median values.

b Median of five-point scale (Excellent/Very good/Good/Fair/Poor).

c Values in parentheses, for binary responses, are the value that B refers to: a positive B value means an independent positive association with change in PPCQ compared to the reference value.

Both Service and Patient Survey question responses showed significant clustering (Table S1). Patient questions that were representative of aspects of service provision are shown in Table 6, with the change in response between 2019 and 2023. Despite mean overall PPCQ falling during this period, there was considerable heterogeneity with several metrics unchanged, and an increase in the proportion of patients reporting a response from the IBD advice line within two working days from 72% to 77% (*P* < .001), a service often used to investigate and manage flare symptoms (eg, diarrhea, pain, or rectal bleeding), but a decrease in discussion of wider life goals and priorities as part of care planning from 30% down to only 24% (*P* < .001).

**Table 6.** Patient assessment of service performance across key aspects of care.

Aspect of care		2019	2023	*P*-value
Access	Wait time from GP referral to hospital appointment <4 weeks	31%	31%	.45
		446/1452	906/2964	
Access	When I contact the IBD advice line, I get a response within 2 working days	72%	77%	<.001
		4228/5851	7667/9958	
Information	The IBD nurse specialists who treat me are knowledgeable about IBD and how to treat the conditions	87%	87%	.69
		6751/7792	10 740/12 414	
Well-being	We discuss my wider life goals and priorities, as part of planning my care	30%	24%	<.001
		2810/9495	3686/15 583	
Empowerment	I have the information and skills to confidently manage everyday symptoms and live as well as possible	64%	64%	.99
		6197/9700	10 057/15 653	

We asked a sample of 17 IBD services who had either the most improvement or most deterioration between 2019 and 2023 to report which factors they felt were most important in the change (Table 7).

**Table 7.** Service-reported factors that they felt were associated with changes in patient-perceived care quality (PPCQ) between 2019 and 2023.

Factors associated with deterioration	Factors associated with improvement
Lack of IBD database following COVID	MDT meetings occurring more often
Increasing out-patient backlog following COVID and fewer clinics following COVID crisis changes that were not restoredLoss of IBD specialist nurses	Starting joint medical/surgical out-patient clinicsImproved elective surgery capacity (with consequent lower emergency surgery rates)
No personnel to staff IBD telephone helpline	Reduced waiting times to start infusion therapy
Retiring IBD gastroenterologist positions not re-appointed	Research activity
High turnover of hospital managers	More IBD specialist nurse roles
Difficulty with staff recruitment (remote area)	New clinic to monitor azathioprine and advanced therapies
	Same day emergency care pathway to reduce emergency admissions
	IBD pharmacist appointment

Abbreviations: MDT, multi-disciplinary team; IBD, inflammatory bowel disease.

## 4. Discussion

Quality improvement is an important goal for all healthcare systems and the majority of people living with IBD have a long-term relationship with their healthcare providers. The IBD UK benchmarking surveys are notable for their breadth of coverage of all aspects of IBD care and the inclusion of both patient and IBD service responses nationwide measured against UK Standards.^3^ Taken together, they provide data which enable service providers at a local level to understand what patients feel about their service and identify areas for improvement. Importantly, they also provide data to support UK-wide campaigns to improve the quality of care for IBD patients. IBD UK has previously shown the importance of patient feedback in assessing service quality, and the emphasis patients place on all aspects of service delivery, including access to care, information provision, communication, empowerment, well-being, and research participation.^4^ We show that adult patient perception of care quality has fallen slightly (although statistically significant due to the large numbers of respondents) over this 4-year period. These data, however, show that the change differs according to the PPCQ level in 2019. Although 2023 PPCQ scores fell in higher performing centers in 2019, PPCQ increased in 2023 in the lower performing centers in 2019.

The demographics of patients responding to the 2023 Patient Survey differed from the IBD population in the UK, with more female respondents, more with Crohn’s disease, more with active disease, and more diagnosed in the past 2 years, mirroring 2019 findings.^4^ Despite attempts to reach ethnic minority groups, these populations are still under-represented in the Patient Survey. Office for National Statistics population data from 2021 for England and Wales report 81.7% in the high-level category of White people. The next most common high-level category is Asian, Asian British, or Asian Welsh people (9.3%), and 2.5% are Black people (including Caribbean and African).^13^ The incidence of IBD in the UK is now as high or higher in Asian people than in White people.^14^ Minority ethnic groups, however, are known to be under-represented in IBD research. A study in over 35 000 IBD patients in the NIHR IBD BioResource database showed that South Asian people made up 3.4%, other Asian groups 0.58%, and Black people 0.47%, with White people 91.03%.^15^ There were 2.4% Asian people and 1% Black people in the present study. The low numbers may relate to language differences, cultural attitudes to health, access to electronic media, or mistrust in healthcare systems, and are widely reported in the UK in relation to research and future research—surveys will need to attempt to address this gap.^16–18^

Patients reported several aspects of care to be highly important: access to treatment, information provision, communication, patient empowerment, well-being support, and research whilst also describing that improvements across all these domains are needed. Both Patient and Service Survey responses were associated with PPCQ, but it was notable that in all but one instance service responses were more positive than patient responses (where comparable questions were compared). The differences were not large, but covered all aspects of care studied, being similar both in more objective measures such as waiting times for assessment, and in subjective areas such as communication and information provision. The reasons for this trend are likely to be multiple. The respondent population was over-represented by those with more active disease. It is possible that patients with negative perceptions of their care had more incentive to participate and may also be over-represented, or patients may more readily recall negative aspects of care. Although a median of 53 patients reported on each IBD service, this is only a small proportion of patients seen, and biases may result. IBD clinicians gave their qualitative assessment of service quality and may conversely recall the more positive aspects of care, and probably considered the full range of patients, many with less challenging care needs. Clinicians were not asked to collect large amounts of data (such as waiting times), as the work required may have prevented service participation, and management support to provide disease-specific waiting times is generally lacking. There is clearly a role for clinicians to provide information about service structure and organization (eg, numbers of staff, provision of joint clinics, or other specialist services) that patients may not be aware of. The differences suggest that more objective measures are also needed. The British Society of Gastroenterology IBD key performance indicators (KPIs) can provide objective data on time to diagnosis and treatment, quality of treatment, and quality of follow-up.^19^

The small but significant fall in patients’ overall view of their care from 2019 to 2023 may relate to the COVID-19 pandemic, with well-documented challenges to healthcare delivery in the UK, as in the rest of the world. The comments from service providers show that IBD services were significantly impacted. Staffing levels fell, and access to endoscopy, elective surgery, and even fecal calprotectin were curtailed.^10^ There were nationwide adaptions to service delivery, with a significant increase in remote consultations.^20^ Many of these changes have continued: remote consulting can be more efficient for services and may be preferred by managers, and many patients find it convenient. However, there are hazards for some patients, particularly those with worsening disease, with unexpected complications, and those requiring physical examination. Patients with disability and difficulty using smart phones or computers may not be able to consult online. Some IBD services lost clinic space with COVID re-organization that has not been restored on the assumption that remote consulting is becoming the norm. Many patients, however, value a flexible service where face-to-face appointments are available when needed.^21^ The pandemic is also likely to have prevented services from introducing quality improvement strategies that required time and staff input. Our data show that there was heterogeneity in changes over the 4 years. Different measures of waiting time access were either unchanged (time to hospital appointment at diagnosis) or improved (access with IBD flare). This could be due to management focus on objective waiting time targets. Information provision and empowerment were unchanged but support for well-being fell. These aspects of care may have been reduced due to staffing shortages, despite our data clearly showing that patients value information provision, communication, empowerment, and well-being support just as highly as waiting times.

More generally during the 4-year period, the NHS experienced severe financial constraints with less access to primary care, fewer emergency hospital assessments, and longer waits for elective surgery. The increase in availability of advanced therapies also puts strain on infusion services, homecare, and monitoring teams, and generates more unmet need for additional specialist IBD nurses and other clinicians to support patients. In this context, a more marked deterioration in PPCQ might have been anticipated, and it is also clear that some aspects of service did improve during this time. Similar findings have been reported more generally for patients with long-term conditions by the National Voices charity collaboration, working in conjunction with Future Health (a public policy research center). They found that numbers of people saying that they are either “very” or “fairly” confident of being able to manage their condition has fallen by 5.4% (from 83.6% in 2019).^22^

Our data show that services with higher PPCQ in 2019 were more likely to have falling scores in 2023, and those with poorer 2019 scores improved. The decline in PPCQ scores may reflect a broader downward trend in public satisfaction with NHS services, as evidenced by the British Social Attitudes (BSA) survey. In 2023, only 24% of the 3374 respondents reported being satisfied with the NHS—the lowest level since the survey began in 1983—with satisfaction falling further to 21% in 2024.^23^^,^^24^ Similar service pressures and challenges in meeting KPIs have been observed across gastroenterology services. The biennial national census of the Joint Advisory Group on Gastrointestinal Endoscopy (JAG) reported a decline in the proportion of NHS services meeting waiting time targets for suspected cancer, routine, and surveillance procedures—from 40.9% in 2019 to 21.9% in 2023.^25^ This decline occurred despite NHS endoscopy services operating at 110% of pre-pandemic activity levels, with staff shortages identified as the most cited factor contributing to delays.

The European Crohn’s and Colitis Organisation (ECCO) published standards in 2020.^26^ The ECCO E-QUALITY project has reported on the gap between Standards and service-reported aspects of care (Structure and Process measures).^27^ ECCO has not as yet published PREMs regarding service quality, and this may relate to the challenges of obtaining data from numerous countries with different languages and cultures. However, ECCO emphasizes the importance of holistic care in their REACH Strategy (see ECCO website: https://ecco-ibd.eu/about-ecco/strategy). In Spain a patient-reported measure of quality of care has been devised (the IQCARO Quality of Care Decalogue) and evaluated in 788 patients across 183 centers.^28^ As with our study, scores were significantly higher in male patients, older patients, those with longer disease duration, and in patients reporting well-controlled disease. In multivariable analysis of 640 online responses, factors linked to higher quality of care scores included being employed, receiving care from an IBD-specialist gastroenterologist, having well-controlled disease, and having fewer unscheduled visits. Another Spanish study evaluated patient experience of IBD specialist nurse support in 80 patients with at least 12 months of experience of living with an IBD diagnosis.^29^ It highlighted the value patients place on specialist nurse support, particularly when a nurse consultation took place after initial diagnosis. This is in keeping with our data, showing the vital role specialist nurses have in providing a contact point for patients needing urgent help. The Spanish study, like ours, shows the value of addressing all aspects of patients’ daily life and environment. The value of management by both doctor and nurse in achieving high-quality care was emphasized. An Italian study from 2017 compared 936 patients’ views and 36 physicians’ views of the quality of IBD care (QUOTE-IBD and Doctor’s QUOTE-IBD questionnaires).^30^ They showed that overall quality improvement scores from physicians were significantly lower than those from patients. In “continuity of care,” however, physicians significantly overestimated quality, compared with patients, whilst underrating their performance in other areas such as “Costs,” “Accessibility,” and “Competence.”

There are a number of strengths in our study, not least the large number of patients and centers that participated but also comparing two distinct periods. The surveys were designed collaboratively by specialists and patients. Patient Surveys were promoted both by IBD clinics and through the Crohn’s & Colitis UK website and social media. We acknowledge some limitations. For pragmatic reasons, service responses were clinician estimates of results and not based on prospective or retrospective evidence. The study under-represented ethnic minority groups, and over-represented females. It is possible that the increased female numbers in 2023 compared to 2019 could have been a contributor to the fall in PPCQ, as females gave lower PPCQ scores in the 2023 and 2019 surveys.^4^ The multilevel, multivariable model of patient and service factors affecting PPCQ did not, however, show a significant impact of sex (albeit with lower analysis numbers). Small numbers meant that we could not determine whether PPCQ differed by ethnic group. The COVID-19 pandemic made it difficult to assess whether service improvement did occur between 2019 and 2023, and future surveys are planned at 4-year intervals, which may make it easier to evaluate trends over time.

Future studies should include objective patient outcome measures alongside patient surveys of service quality and service-reported structure and processes. Further work is needed to encourage and support local IBD services to use their own data to support targeted quality improvement projects.

## 5. Conclusion

This study utilized high-volume patient assessment of quality of care delivery in IBD—a complex long-term health condition—alongside data from service providers. Whilst it is important to recognize that patient factors, including disease activity at the time of survey completion, sex, and age, affect their perception of overall care quality, the data underscore the importance of assessing lived experience and the care quality perception gap between patients and service providers. Patients value qualitative aspects such as communication and information delivery that are often undervalued in specialist-led assessments. Regular benchmarking including PREMs should be used to drive and assess service-level, national, and international quality improvement initiatives.

## Supplementary Material

jjag005_Supplementary_Data

## Data Availability

Anonymized data from IBD Benchmarking are available at https://ibduk.org/ibd-benchmarking-tool.
