# Supplementary material for: Comparative analysis of inflammatory bowel disease (IBD) patient- and service-reported quality of care using 2019 and 2023 UK benchmarking data from more than 26 000 adult patient respondents and 154 IBD services
Source: J Crohns Colitis. 2026 Jan 23;20(3):jjag005. doi: 10.1093/ecco-jcc/jjag005 (PMC13017129; doi:10.1093/ecco-jcc/jjag005)
Supplement: jjag005_Supplementary_Data [file jjag005_supplementary_data.docx]

## Supplement

## Clustering of survey responses

Principal component analysis was used to compare similarity in responses across a range of questions for Patient and Service Surveys separately. They showed clustering into groups (**Supplementary Table 1a-c**). The yes/no questions are analysed separately to those with multiple responses (Service Survey). Values presented are essentially a correlation R value (0-1).

## Supplementary Table 1a Patient Survey PCA clustering

|  | Patient priorities | Communication  /information | Access | Audit/ Research |
| --- | --- | --- | --- | --- |
| How long did you have symptoms before you first saw a healthcare professional about them? A healthcare professional is a doctor, nurse, or occasionally pharmacist or dietitian | 0.14 | -0.19 | **0.66** | 0.05 |
| How long did it take from the time you first spoke to a healthcare professional about your symptoms, to the time you were referred to be seen in hospital? Being referred is when your GP asks a hospital specialist to see you about your symptoms, not when they actually see you | -0.05 | 0.08 | **0.77** | 0.01 |
| How long was the wait between being referred to hospital and a diagnosis being made? | 0.05 | 0.07 | **0.72** | -0.05 |
| How long after your diagnosis were you able to start your treatment? Treatment in this situation could have been drug or dietary therapy (e.g. exclusive enteral nutrition). If you did not need any specific treatment, or an operation then choose 'Not applicable'. | 0.13 | -0.06 | **0.5** | -0.05 |
| Do you have access to an IBD nurse specialist? An IBD nurse specialist is a nurse who is specifically trained to work with patients who have Crohn's, colitis and other forms of IBD. | -0.02 | **0.25** | 0.02 | 0.08 |
| When I contact the IBD advice line, I get a response within 2 working days | 0.32 | **0.45** | 0.01 | -0.33 |
| The IBD nurse specialists who treat me are knowledgeable about IBD and how to treat the conditions | 0.19 | **0.57** | -0.05 | -0.22 |
| My GP is knowledgeable about IBD and is helpful | -0.25 | **0.58** | 0.29 | 0.03 |
| I have the information and skills to confidently manage everyday symptoms and live as well as possible | 0 | **0.76** | 0.06 | 0.05 |
| I am given enough information about treatment and care options to let me make an informed decision. This includes information about possible benefits and side-effects | 0.12 | **0.76** | -0.02 | 0.07 |
| We discuss my wider life goals and priorities, as part of planning my IBD care | 0.06 | **0.71** | 0.01 | 0.17 |
| I am involved as much as I want to be in decisions about my care and treatment | 0.14 | **0.74** | -0.01 | -0.01 |
| These reviews give me the opportunity to discuss what matters most to me | 0.03 | **0.72** | -0.04 | 0.07 |
| Improvement needed in Access (quickly/easy to make contact/convenient for you in timing and location of appointments) | **0.84** | 0.05 | 0.04 | -0.06 |
| Improvement needed in Information (to help you understand your condition and treatment options) | **0.76** | 0.14 | 0.03 | 0.03 |
| Improvement needed in Communication (between you and your team and also how they work  together) | **0.8** | 0.12 | 0.05 | -0.03 |
| Improvement needed in Empowerment (enabling you to make choices and manage your own  condition where possible) | **0.8** | 0.04 | 0 | 0.05 |
| Improvement needed in Well-being support (support from the IBD team for my overall physical and  mental health and ability to manage my life day to day) | **0.82** | 0.01 | -0.01 | -0.02 |
| Improvement needed in Research (involvement or information about IBD research  trials/surveys/collection of data going on locally or nationally) | **0.69** | -0.22 | 0.04 | 0.24 |
| Do you have a personalised written care plan? | 0.13 | 0.15 | -0.2 | **0.41** |
| In the last 12 months, have you had the opportunity to give feedback specifically on your IBD care in any of the following ways? Please tell us even if you didn't participate. | 0.07 | 0.14 | -0.05 | **0.62** |
| In the last 12 months, have your IBD team offered you any of the following opportunities to take part in clinical trial/s and/or IBD research? Research can be taking part in surveys, focus groups, providing your clinical data for research studies, as well as clinical trials | 0.05 | 0.11 | 0.06 | **0.71** |

## Supplementary Table 1b Service Survey PCA clustering 1

|  | Component 1 | Component 2 |
| --- | --- | --- |
| Audits of different aspects of the IBD service are carried out at least annually | **0.6** | -0.08 |
| There is a locally agreed policy for steroid use in IBD | **0.69** | 0 |
| Home parenteral nutrition is available locally for intestinal failure | **0.6** | -0.09 |
| There is locally agreed policy for referral of suspected IBD between primary and secondary care | **0.57** | 0.03 |
| All newly diagnosed IBD patients are seen in a dedicated IBD clinic | **0.63** | 0.14 |
| There is an agreed departmental process for assessment of newly diagnosed IBD patients that includes nutritional status / bone health / baseline infection screen | **0.76** | -0.08 |
| Do combined medical and surgical clinic consultations occur? | -0.05 | **0.71** |
| There is a surgery specific IBD nurse available to support patients undergoing IBD operations | -0.11 | **0.65** |
| Most patients are seen by an IBD nurse specialist during hospital admission | -0.03 | **0.39** |
| Copies of clinic letters are routinely sent to IBD patients | 0.04 | **0.55** |
| IBD patients are involved in service development, e.g. through an IBD patient panel or specific project groups | 0.3 | **0.48** |
| Patients are supported to be actively involved in management decisions about their care | 0.41 | **0.43** |

## Supplementary Table 1c Service Survey PCA clustering 2

| Yes/No questions | Component 1 | Component 2 |
| --- | --- | --- |
| Does the IBD service have a leadership team including a senior clinician, IBD nurse specialist and manager? | **0.93** | -0.05 |
| Is your IBD service led by a named gastroenterologist? | **0.71** | 0.18 |
| Faecal calprotectin is available to all GP's for investigation of suspected IBD | 0 | **0.98** |
| Are IBD MDTS supported by clerical staff for minute taking and organisation? | **0.67** | 0.09 |
